# Sedation, temperature and pressure after cardiac arrest and resuscitation—The STEPCARE trial: A statistical analysis plan

**DOI:** 10.1111/aas.70033

**Published:** 2025-04-10

**Authors:** C. B. Kamp, J. Dankiewicz, M. Harboe Olsen, J. Holgersson, M. Saxena, P. Young, V. H. Niemelä, J. Hästbacka, H. Levin, G. Lilja, M. Moseby‐Knappe, M. Tiainen, M. Reinikainen, A. Ceric, J. Johnsson, J. Undén, J. Düring, A. Lybeck, D. Rodriguez‐Santos, A. Lundin, J. Kåhlin, J. Grip, E. Lotman, L. Romundstad, P. Seidel, P. Stammet, T. Graf, A. Mengel, C. Leithner, J. Nee, P. Drúwe, K. Ameloot, M. P. Wise, P. J. McGuigan, A. Ratcliffe, J. Cole, J. White, N. Pareek, G. Glover, R. Handslip, A. Proudfoot, M. Thomas, D. Pogson, T. R. Keeble, A. Nichol, M. Haenggi, M. P. Hilty, M. Iten, C. Schrag, M. Nafi, M. Joannidis, C. Robba, T. Pellis, J. Belohlavek, O. Smid, D. Rob, Y. Arabi, S. Buabbas, C. Yew Woon, Q. Li, M. Reade, A. Delaney, B. Venkatesh, N. Hammond, F. Bass, A. Aneman, A. Stewart, L. Navarra, B. Crichton, D. Knight, A. Williams, J. Tirkkonen, T. Oksanen, T. Kaakinen, S. Bendel, H. Friberg, T. Cronberg, M. B. Skrifvars, N. Nielsen, J. C. Jakobsen

**Affiliations:** ^1^ Copenhagen Trial Unit, Centre for Clinical Intervention Research, The Capital Region Copenhagen University Hospital – Rigshospitalet Copenhagen Ø Denmark; ^2^ Department of Regional Health Research The Faculty of Health Sciences, University of Southern Denmark Odense Denmark; ^3^ Department of Clinical Sciences Lund, Section of Cardiology Skåne University Hospital Scania Sweden; ^4^ Department of Neuroanaesthesiology The Neuroscience Centre, Copenhagen University Hospital – Rigshospitalet Copenhagen Denmark; ^5^ Anesthesiology and Intensive Care Department of Clinical Sciences, Helsingborg Hospital Lund, Lund University Lund Sweden; ^6^ Critical Care Division and Department of Intensive Care Medicine The George Institute for Global Health Sydney NSW Australia; ^7^ St George Hospital Clinical School University of New South Wales Sydney Australia; ^8^ Intensive Care Unit Wellington Hospital Wellington New Zealand; ^9^ Medical Research Institute of New Zealand Wellington New Zealand; ^10^ Australian and New Zealand Intensive Care Research Centre Monash University Melbourne Victoria Australia; ^11^ Department of Critical Care University of Melbourne Melbourne Victoria Australia; ^12^ Department of Anaesthesia and Intensive Care Helsinki University Hospital and University of Helsinki Helsinki Finland; ^13^ Tampere University Hospital Wellbeing Services County of Pirkanmaa and Tampere University, Faculty of Medicine and Health Technology Tampere Finland; ^14^ Department of Clinical Sciences Lund Lund University Lund Sweden; ^15^ Department of Research, Development, Education and Innovation Skåne University Hospital Lund Sweden; ^16^ Department of Neurology and Rehabilitation Skåne University Hospital Lund Sweden; ^17^ Neurology Department of Clinical Sciences Lund, Lund University Lund Sweden; ^18^ Department of Neurology Helsinki University Hospital and University of Helsinki Helsinki Finland; ^19^ University of Eastern Finland Institute of Clinical Medicine Finland; ^20^ Department of Anaesthesiology and Intensive Care Kuopio University Hospital Finland; ^21^ Anaesthesia & Intensive Care Department of Clinical Sciences, Lund University, Skåne University Hospital Malmö Sweden; ^22^ Department of Anaesthesiology and Intensive Care Helsingborg Hospital Helsingborg Sweden; ^23^ Department of Operation and Intensive Care Hallands Hospital Halmstad Sweden; ^24^ Department of Intensive and Perioperative Care Skåne University Hospital, Lund University Lund Sweden; ^25^ Department of Anaesthesia and Intensive Care Skaraborgs Sjukhus Skövde Sweden; ^26^ Department of Anaesthesiology and Intensive Care Medicine Institute of Clinical Sciences, Sahlgrenska Academy, University of Gothenburg Gothenburg Sweden; ^27^ Perioperative Medicine and Intensive Care (PMI) Karolinska University Hospital Stockholm Sweden; ^28^ Department of Physiology and Pharmacology Karolinska Institute Stockholm Sweden; ^29^ Department of Clinical Science, Intervention and Technology Karolinska Institute Stockholm Sweden; ^30^ North Estonia Medical Centre Estonia; ^31^ Department of Anaesthesia and Intensive Care Medicine Division of Emergencies and Critical Care, Oslo University Hospital Oslo Norway; ^32^ Lovisenberg Diaconal University College Oslo Norway; ^33^ Department of Intensive Care Medicine Stavanger University Hospital Stavanger Norway; ^34^ Department of Anaesthesia and Intensive Care Medicine Centre Hospitalier de Luxembourg Luxembourg Luxembourg; ^35^ University Heart Center Lübeck, University Hospital Schleswig‐Holstein Germany; ^36^ German Center for Cardiovascular Research (DZHK), Partner Site Hamburg/Lübeck/Kiel Germany; ^37^ Department of Neurology and Stroke University Hospital Tuebingen Germany; ^38^ Hertie Institute of Clinical Brain Research Germany; ^39^ Charité – Universitätsmedizin Berlin, Corporate Member of Freie Universität and Humboldt‐Universität zu Berlin Department of Neurology Berlin Germany; ^40^ Department of Nephrology and Medical Intensive Care Charité ‐ Universitaetsmedizin Berlin Berlin Germany; ^41^ Department of Intensive Care Medicine Ghent University Hospital Ghent Belgium; ^42^ Department of Cardiology Ziekenhuis Oost‐Limburg Genk Belgium; ^43^ Adult Critical Care University Hospital of Wales Cardiff UK; ^44^ Wellcome‐Wolfson Institute for Experimental Medicine Queen's University Belfast UK; ^45^ Regional Intensive Care Unit Royal Victoria Hospital Belfast UK; ^46^ Leeds General Infirmary, The Leeds Teaching Hospitals NHS Trust Leeds UK; ^47^ Critical Care University Hospital of Wales Cardiff UK; ^48^ Centre for Healthcare Evaluation, Device Assessment and Research Cardiff and Vale University, Health Board Cardiff UK; ^49^ Department of Cardiology King's College Hospital NHS Foundation Trust London UK; ^50^ School of Cardiovascular and Metabolic Medicine & Sciences, BHF Centre of Excellence King's College London UK; ^51^ Department of Critical Care Guy's and St Thomas NHS Foundation Trust London UK; ^52^ St George's University Hospital NHS Foundation Trust London UK; ^53^ Department of Perioperative Medicine Barts Heart Centre, St Bartholomew's Hospital London UK; ^54^ University Hospitals, Bristol and Weston UK; ^55^ Department of Critical Care Portsmouth University Hospitals Trust Portsmouth UK; ^56^ Essex Cardiothoracic Centre, MSE NHSFT Essex UK; ^57^ Anglia Ruskin School of Medicine & MTRC, ARU Essex UK; ^58^ University College Dublin Clinical Research Centre at St Vincent's University Hospital University College Dublin Dublin Ireland; ^59^ The Australian and New Zealand Intensive Care Research Centre, Monash University Melbourne Australia; ^60^ The Alfred Hospital Melbourne Australia; ^61^ Institute of Intensive Care Medicine University Hospital Zurich Zurich Switzerland; ^62^ Department of Intensive Care Medicine Inselspital University Hospital Bern Bern Switzerland; ^63^ Klinik für Intensivmedizin, Kantonsspital St. Gallen St. Gallen Switzerland; ^64^ Istituto Cardiocentro Ticino Lugano Switzerland; ^65^ Division of Intensive Care and Emergency Medicine Department of Internal Medicine, Medical University Insbruck Innsbruck Austria; ^66^ IRCCS Policlinico San Martino Genova Italy; ^67^ Dipartimento di Scienze Chirurgiche Diagnostiche Integrate University of Genova Italy; ^68^ Anaesthesia and Intensive Care Pordenone Hospital Azienda Sanitaria Friuli Occidentale Italy; ^69^ 2nd Department of Internal Medicine, Cardiovascular Medicine General University Hospital Prague Czech Republic; ^70^ 1st Faculty of Medicine, Charles University Prague Czech Republic; ^71^ Institute for Heart Diseases Wroclaw Medical University Wrocław Poland; ^72^ King Saud bin Abdulaziz University for Health Sciences and King Abdullah International Medical Research Center Riyadh Saudi Arabia; ^73^ Department of Anaesthesia, Critical Care and Pain Medicine Jaber Alahmad Alsabah Hospital Kuwait; ^74^ Tan Tock Seng Hospital Singapore Singapore; ^75^ Yong Loo Lin School of Medicine National University of Singapore Singapore Singapore; ^76^ Lee Kong Chian School of Medicine Nanyang Technological University Singapore Singapore; ^77^ The George Institute for Global Health University of New South Wales Sydney Australia; ^78^ Medical School University of Queensland Brisbane Australia; ^79^ Malcolm Fisher Department of Intensive Care Medicine Royal North Shore Hospital St Leonards Australia; ^80^ Northern Clinical School, Sydney Medical School University of Sydney Camperdown New South Wales Australia; ^81^ The George Institute for Global Health Sydney New South Wales Australia; ^82^ Critical Care Program The George Institute for Global Health, UNSW Sydney Australia; ^83^ Royal North Shore Hospital Sydney Australia; ^84^ Intensive Care Unit Liverpool Hospital, South Western Sydney Local Health District Sydney New South Wales Australia; ^85^ South Western Clinical School University of New South Wales Sydney New South Wales Australia; ^86^ The Ingham Institute for Applied Medical Research Sydney New South Wales Australia; ^87^ Liverpool Hospital, South Western Sydney Local Health District Liverpool New South Wales Australia; ^88^ Department of Intensive Care Christchurch Hospital New Zealand; ^89^ Middlemore Hospital Auckland New Zealand; ^90^ Intensive Care Unit Tampere University Hospital Finland; ^91^ Research Unit of Translational Medicine, Research Group of Anaesthesiology, Medical Research Center Oulu Oulu University Hospital and University of Oulu Oulu Finland; ^92^ OYS Heart, Oulu University Hospital MRC Oulu and University of Oulu Oulu Finland; ^93^ Intensive and Perioperative Care Skåne University Hospital Malmö Sweden; ^94^ Anesthesia and Intensive Care Department of Clinical Sciences Lund, Lund University Sweden

**Keywords:** blood pressure, cardiac arrest, sedation, statistical analysis, STEPCARE trial, temperature

## Abstract

**Background:**

Basic management for patients who have suffered a cardiac arrest and are admitted to an intensive care unit (ICU) after resuscitation includes setting targets for blood pressure and managing sedation and temperature. However, optimal targets and management are unknown.

**Methods:**

The STEPCARE (Sedation, Temperature and Pressure after Cardiac Arrest and Resuscitation) trial is a multicenter, parallel‐group, randomized, factorial, superiority trial in which sedation, temperature, and blood pressure strategies will be studied in three separate comparisons (SED‐CARE, TEMP‐CARE, and MAP‐CARE). The trial population will be adults admitted to intensive care who are comatose after resuscitation from out‐of‐hospital cardiac arrest. The primary outcome will be all‐cause mortality, and the secondary outcomes will be poor functional outcome (modified Rankin Scale 4–6), Health‐Related Quality of Life using EQ‐VAS, and specific serious adverse events in the intensive care unit predefined for each trial. All outcomes will be assessed at 6 months after randomization. The prognosticators, outcome assessors, statisticians, data managers, steering group, and manuscript writers will be blinded to treatment allocation. This statistical analysis plan includes a comprehensive description of the statistical analyses, handling of missing data, and assessments of underlying statistical assumptions. Analyses will be conducted according to the intention‐to‐treat principle, that is, all randomized participants with available data will be included. The analyses will be performed independently by two statisticians following the present plan.

**Conclusion:**

This statistical analysis plan describes the statistical analyses for the STEPCARE trial in detail. The aim of this predefined statistical analysis plan is to minimize the risk of analysis bias.

## 1 BACKGROUND

Critical care management for patients who have suffered an out‐of‐hospital cardiac arrest (OHCA) who require treatment in an intensive care unit (ICU) includes setting goals and targets for the use of medications and devices that influence vital signs and other physiological parameters for the purpose of reducing ischemia–reperfusion injury. Sedation, temperature management, and blood pressure strategies are common examples of how clinicians deploy therapies in this patient population; however, optimal targets are unknown.^1^, ^2^

The STEPCARE trial is a multicenter, parallel‐group, non‐commercial, randomized, factorial, superiority trial in which sedation, temperature, and blood pressure strategies will be studied. The STEPCARE trial is a continuation of the collaborations from the previous Target Temperature Management after out‐of‐hospital cardiac arrest trial (TTM),^3^ Targeted Hypothermia versus Targeted Normothermia after Out‐of‐hospital Cardiac Arrest trial (TTM2),^4^ and the Carbon dioxide, Oxygen and Mean arterial pressure After Cardiac Arrest and Resuscitation trial (COMACARE).^5^

Through STEPCARE, this international collaboration aims to improve the evidence for critical care in cardiac arrest patients. Here we describe the statistical analysis plan that will outline the statistical analyses for the STEPCARE trial in detail.

## 2 METHODS

The STEPCARE trial is registered at clinicaltrials.gov (NCT05564754, 2022‐10‐03). We describe the STEPCARE trial design and its intervention in detail in three separate protocols. In brief, the trial population will be adults (≥18 years) admitted after OHCA with the return of spontaneous circulation (ROSC). Patients will be eligible for enrolment if they meet all the following inclusion criteria and none of the exclusion criteria:

### 2.1 Inclusion criteria

- Out‐of‐hospital cardiac arrest
- A minimum of 20 min without chest compressions (20 min of spontaneous circulation without the need for chest compressions)
- Unconsciousness defined as not being able to obey verbal commands (Full Outline of UnResponsiveness (FOUR) score motor response of <4) or being intubated and sedated because of agitation after sustained ROSC.
- Eligible for intensive care without restrictions or limitations
- Inclusion within 4 h of ROSC

### 2.2 Exclusion criteria

- Trauma or hemorrhage (including gastrointestinal bleeding) as presumed cause of arrest
- On Extracorporeal Membrane Oxygenation (ECMO) prior to randomization
- Pregnancy
- Suspected or confirmed intracranial hemorrhage
- Previously randomized in the STEPCARE trial

### 2.3 Randomization and blinding

Trial sites will have access to an internet‐based application for immediate randomization to ensure adequate allocation concealment and sequence generation. Each patient will be assigned to three trials simultaneously. Randomization will be performed with permuted blocks of varying size, stratified for trial site only. Investigators will be blinded to the block sizes. The team treating the patient (physicians, nurses, and others) will not be blinded to the allocation group due to the inherent difficulty in blinding the interventions (sedation, temperature, and arterial pressure). Measures will be taken to ensure that the information about allocation will not be disseminated beyond the immediate group of caregivers responsible for patient care. The clinicians performing neuroprognostication, outcome assessors, statisticians, data managers, steering group, and manuscript writers will be blinded to treatment allocation.

### 2.4 Trial interventions

The STEPCARE trial includes three separate comparisons.

### 2.5 The SED‐CARE trial

In the SED‐CARE trial, participants are randomized to either continuous deep sedation or minimal sedation. Initially, patients could be sedated to ensure safe transport, imaging, coronary angiography, and other invasive procedures. Following randomization, the Richmond Agitation‐Sedation Scale (RASS) score and FOUR score motor response will be collected every 4 h. For patients randomized to continuous deep sedation, the target will be a RASS from −4 to −5 upon admission to the ICU and continued sedation until 36 h after randomization. In patients randomized to minimal sedation, sedative agents should be used only as needed for clinical care. After the 36‐h intervention period, the sedation strategy for both groups will be at the discretion of the treating physician as needed for clinical care.

### 2.6 The TEMP‐CARE trial

In the TEMP‐CARE trial, participants are randomized to fever management with or without a device. The intervention period will commence immediately after randomization. Core body temperature will be continuously monitored (preferentially via a bladder catheter, but an alternative core temperature site such as esophagus or blood will be allowed). For participants allocated to fever management with a device, temperature management devices will be started to achieve a core body temperature of ≤37.5°C if the temperature reaches the trigger of ≥37.8°C within 72 h of randomization. For participants allocated to fever management without a device, fever will be managed as per standard treatment for any critically ill patient in the ICU, including exposure and pharmacological agents, where considered clinically necessary. The temperature intervention will last until 72 h after randomization, or until extubation, whichever occurs first. If the participant regains consciousness and does not require invasive ventilation prior to 72 h after randomization, then temperature management is at the discretion of the treating clinician. After discharge from the intensive care unit, temperature management is also at the discretion of the treating clinician.

### 2.7 The MAP‐CARE trial

In the MAP‐CARE trial, participants are randomized to a mean arterial pressure (MAP) target of either >85 mmHg or >65 mmHg. All patients must have invasive monitoring of blood pressure. The intervention period will commence immediately after randomization, but titration of vasopressors to the MAP target may be delayed until the patient has completed the required diagnostic work‐up (e.g., computed tomography scan or coronary angiography) and is admitted to intensive care. The means to achieve the desired MAP target will be up to the treating clinician, but the primary recommendation is to titrate a vasopressor unless the patient is hypovolemic, in which case judicious doses of fluids should be prescribed. The MAP intervention will last until 72 h after randomization, or until extubation, whichever occurs first. After 72 h, or earlier if the participant regains consciousness and is extubated, and after discharge from intensive care, the management of MAP will be at the discretion of the treating physician.

### 2.8 Outcomes

The primary assessment time point will be 6 months after randomization for all outcomes. The outcomes are defined as primary, secondary, and exploratory. The sample size is based on the primary outcome, and our primary conclusions will be based on the results of the primary outcome.

Primary outcome

- All‐cause mortality

Secondary outcomes

- Proportion of participants with a poor functional outcome measured at 6 months after randomization in either one of two ways (hierarchical order):
  - Using the modified Rankin Scale (mRS) with mRS 4–6 indicating a poor outcome (dichotomized)
  - OR if a mRS score cannot be assigned using the standardized assessment, a poor outcome (yes/no) will be based on whether the patient is dependent on others for basic activities of daily living (moving indoors, eating, dressing, personal hygiene)
- Proportion of patients with a predefined serious adverse event in the index intensive care stay (defined differently for each trial):
  - In *SED‐CARE*: death, sepsis or septic shock (Sepsis‐3 criteria),^6^ arrhythmia requiring defibrillation, cardioversion or chest compressions, venous thromboembolism, reintubation, or unplanned extubation
  - In *TEMP‐CARE*: death, sepsis or septic shock (Sepsis‐3 criteria),^6^ arrhythmia requiring defibrillation, cardioversion or chest compressions, venous thromboembolism, moderate or severe bleeding (GUSTO criteria)^7^
  - In *MAP‐CARE*: death, arrhythmia requiring defibrillation, cardioversion, or chest compressions, moderate or severe bleeding (GUSTO criteria),^7^ acute kidney injury with renal replacement therapy, limb ischemia requiring radiological or surgical intervention, or gut ischemia demonstrated at laparotomy/laparoscopy or clinically suspected based on abdominal CT‐scan
- Patient‐reported Health‐Related Quality of Life (HRQoL) using EQ‐5D‐5L measured by EQ‐VAS.^8^

### 2.9 Exploratory outcomes

- mRS (ordinal score)
- Ventilator‐free days within 30 days
- Hospital/institution‐free days alive within the first 30 days

### 2.10 Sample size and power estimations

Based on the results of the TTM trial, TTM2 trial, and information from the International Cardiac Arrest Registry (INTCAR),^9^ we estimate that within 6 months after randomization, approximately 60% of the participants will die. We hypothesize that 6 months after randomization, 54.4% of the participants will die in the intervention groups, corresponding to a relative risk reduction of 9% (relative risk, RR = 0.91). With an acceptable risk of type‐1 error of 5% and an acceptable risk of type‐2 error of 10% (power of 90%), a total of 3278 participants (1639 in each pairwise comparison) would be required.

We plan to investigate the possible effects of adjustment for site and possible interactions between interventions using simulations.^10^ Preliminary simulations suggest that if there are interactions between the trial interventions on outcomes, it would be necessary to increase the sample size. The STEPCARE trial is being conducted with the assumption that there will be no such interactions between the trial interventions on outcomes. However, to accommodate the possibility of minor interactions, an increase in the sample size (5%) has been chosen to account for this. The combined effect of withdrawn consent and missing data on the primary outcome in the TTM2 trial resulted in a sample size reduction of approximately 2%. We assume a similar result in the current trial (1.8%). Considering the above‐mentioned factors, we will increase the sample size by 6.8% and recruit a total of 3500 participants.

We estimated the statistical power of all secondary outcomes.^11^ With an estimated sample size of 3500 participants in total, the functional outcome measure (dichotomized mRS) has a power of 98% to detect a relative risk of 0.90 for a poor functional outcome (mRS 4–6) in the experimental group, with 65% of cases in the control group experiencing a poor functional outcome.

For the predefined serious adverse events, we performed simulations to assess the incidences for the three serious adverse event outcomes. We had estimated incidences for arrhythmia, sepsis, and bleeding, but not for unplanned extubation, reintubation, and venous thromboembolism.^12^, ^13^ We also considered aggregate data from previous meta‐analyses and trials, and these numbers roughly corresponded to the simulations:^3^, ^4^, ^14^, ^15^, ^16^

- In SED‐CARE: We estimate a power of 92% to detect a relative risk reduction or increase of 9% for the predefined serious adverse events with 61% of cases in the control group.
- In TEMP‐CARE: We estimate a power of 92% to detect a relative risk reduction or increase of 9% for the predefined serious adverse events with 63% of cases in the control group.
- In MAP‐CARE: We estimate a power of 91% to detect a relative risk reduction or increase of 10% for the predefined serious adverse events with 57% of cases in the control group.

The anticipated intervention effects in the above‐mentioned sample size and the power estimations correspond to an absolute risk reduction of 5.6%.

We estimate a power of >90% to detect a difference in 5 points on the EQ‐5D‐5L VAS scale, based on a mean value of 75 in the control group and a standard deviation of 20 points.^4^

### 2.11 General analysis principles

We will conduct all analyses according to the intention‐to‐treat principle (ITT), that is, all randomized participants with available data will be included in all analyses.

We will assess if the statistical and clinical significance thresholds are crossed (Bayes factor calculations will be reported in supplementary material).^17^ Assessment of clinical significance will be based on the anticipated intervention effects used in the sample size and power estimations.^17^ Our primary conclusion will be based on the primary outcome, so all tests of statistical significance (including subgroup analyses) will be two‐sided with a threshold of 5%.^17^ No conclusions will be drawn based on secondary outcomes.

It is generally acknowledged that regression analyses ought to be adjusted for the stratification variables used in the randomization.^18^, ^19^, ^20^ Hence, when analyzing each of the three trials, we will adjust all analyses for ‘site’ and the allocated intervention in the two other trials (e.g., the SED‐CARE outcomes will be adjusted for ‘site’ and the allocated TEMP‐CARE and MAP‐CARE interventions). We will assess whether there are significant interactions between trial interventions (for details, see Section 2.14).

We will perform the following subgroup analyses:

- Age (higher or lower than the median)
- Sex (male/female)
- Cardiopulmonary resuscitation by bystander (yes/no)
- Initial rhythm (shockable vs. non‐shockable)
- Time to ROSC (higher or lower than the median)
- Circulatory status on admission (presence or absence of circulatory shock diagnosed by the treating physician)
- Baseline risk of poor neurologic outcome (Miracle2‐score: low risk (0–2), medium (3–5), and high (6–10).^21^)
- Presumed cause of arrest at randomization (cardiac vs. others)
- Chronic hypertension as a comorbidity

We plan to reanalyze all three trials using Bayesian statistics. Separate statistical analysis plans will be published.

### 2.12 Statistical analyses

#### 2.12.1 Outcomes

We will analyze the primary outcome as a binary variable (alive or dead). We will analyze the secondary outcomes as follows: (I) We will evaluate the functional outcome by dichotomizing the modified Rankin scale (0–3 vs. 4–6), or if this is missing, dependent versus independent in basic activities of daily life (moving indoors, eating, dressing, personal hygiene). (II) We will analyze serious adverse events as a binary variable (one or more versus no serious adverse event). Additionally, we will present each specific serious adverse event separately by risk ratios and 95% confidence intervals. (III) We will analyze HRQoL by EQ‐VAS scale as a continuous variable (0–100).

#### 2.12.2 Analysis of dichotomous data

We will present dichotomized outcomes as proportions of participants in each group with the event, as well as relative risks with 95% confidence intervals. We will analyze dichotomous outcomes using mixed effects generalized linear models using a log‐link function with ‘site’ as a random intercept using an exchangeable covariance matrix, and we will include the allocated intervention in the two other trials as fixed effects. If the analysis does not converge, we will use mixed effects logistic regression to analyze dichotomized data and relative risks will be obtained using either G computation in R (R Core Team, Vienna, Austria) or the NLCOM command in Stata 18 (StataCorp, College Station, TX, USA).

#### 2.12.3 Analysis of continuous data

We will present continuous outcomes as means and standard deviations for each group, along with 95% confidence intervals for the means of the groups and the mean differences between the groups. We will analyze continuous outcomes using mixed effects linear regression with ‘site’ as a random intercept using an exchangeable covariance matrix, and we will include the allocated intervention in the two other trials as fixed effects. We expect that a large proportion of the participants will die before the assessment of HRQoL. When assessing health‐related HRQoL, we will, therefore, in a secondary analysis, impute a ‘0’ for all participants who died. We will analyze these data using the stratified Wilcoxon test stratified for the other interventions.

#### 2.12.4 Analysis of count data (ventilator‐free days, hospital/institution‐free days, and mRS (ordinal score))

We will present count data as means, mean differences, and 95% confidence intervals or medians, interquartile ranges, and 95% confidence intervals (bootstrapping) depending on the observed distribution. We will analyze count data using the stratified Wilcoxon test stratified for the other interventions.

We will present mock tables for the three comparisons separately in each protocol. This includes baseline tables, results tables, survival curves, and graphs/tests illustrating separation between the groups (sedation, temperature, and MAP).

### 2.13 Handling of missing data

In the primary analysis we will include all randomized participants with available data (please see the Section 2.12.3). We will handle missing data according to the recommendation by Jakobsen et al.^22^ In short, we anticipate that the proportion of missing values on main dichotomous outcomes will be less than 5%. We will consider using multiple imputations and present best‐worst and worst‐best case scenarios if it is not valid to ignore missing data.^22^ Best‐worst and worst‐best case scenarios assess the potential range of impact of the missing data for the trial results.^22^ In the best‐worst case scenario, it is assumed that all patients lost to follow‐up in the experimental group had a beneficial outcome (survived, had no poor functional outcome, etc.), and all those with missing outcomes in the control group had a harmful outcome (did not survive, had poor functional outcome, etc.).^22^ Conversely, in the worst‐best case scenario, it is assumed that all patients who were lost to follow‐up in the experimental group had a harmful outcome and that all those lost to follow‐up in the control group had a beneficial outcome.^22^ When continuous outcomes are used, we will define a beneficial outcome as the group mean plus two SDs of the group mean (fixed imputation), and a harmful outcome as the group mean minus two SDs of the group mean (fixed imputation).^22^

We will perform sensitivity analyses assuming that all participants with missing data were either all alive or dead.

### 2.14 Assessments of underlying statistical assumptions

We will systematically assess underlying statistical assumptions for all statistical analyses.^23^, ^24^ In short, we will test for major interactions between the three intervention variables for all primary and secondary regression analyses. We will only consider that there is evidence of an interaction if the interaction is statistically significant (*p* < .01) and if the interaction shows a clinically important effect (based on the anticipated intervention effect used in the sample size, that is, an absolute risk reduction of 5.6%). If it is concluded that the interaction is significant both statistically and clinically, we will reconsider whether the three comparisons should be presented in three separate main articles.^23^, ^24^

#### 2.14.1 Assessments of underlying statistical assumptions for dichotomous outcomes

We will assess if the deviance divided by the degrees of freedom is significantly larger than 1 to assess for relevant overdispersion. Overdispersion is the presence of greater variability (statistical dispersion) than expected in a data set based on a given statistical model. Relevant overdispersion will be handled using a maximum likelihood estimate of the dispersion parameter. To avoid analytical problems with few events or problems with all participants dying at a given site, we have only included sites planning to randomize at least 30 participants. We will consider pooling data from smaller sites if the number of participants is too low.^24^

#### 2.14.2 Assessments of underlying statistical assumptions for linear regression

We will visually inspect quantile–quantile plots of the residuals to assess if the residuals are normally distributed and use residuals plotted against covariates and fitted values to assess for homogeneity of variances.^25^, ^26^ If the plots show deviations from the model assumptions, we will consider transforming the outcome, for example, using log transformation or square root and/or using robust standard errors.^24^, ^25^, ^26^

### 2.15 Statistical reports

Blinded data on all outcomes will be analyzed by two independent statisticians.^24^ Two independent statistical reports will be sent to the chief principal investigator and will be shared with the steering group and author group, and if there are discrepancies between the two primary statistical reports, then possible reasons for those will be identified and the steering group will decide which is the most correct result. We will prepare one final statistical report and publish all three statistical reports as supplementary material.^24^

## 3 DISCUSSION

The aim of this predefined statistical analysis plan is to minimize the risk of biased data‐driven analyses. This statistical analysis plan describes the analyses for the three STEPCARE trials: SED‐CARE, TEMP‐CARE, and MAP‐CARE in detail.

This statistical analysis plan has several strengths. The methodology is predefined and detailed to avoid data‐driven analyses and risks of outcome reporting bias. All primary and secondary outcomes are patient‐centered, and our primary conclusions will be based on the primary outcome to limit problems with multiplicity.^17^ We will analyze data according to the intention‐to‐treat principle and, if necessary, use multiple imputation and best‐worst/worst‐best case scenarios to assess the potential impact of the missing data on the results.^22^ Furthermore, we plan to systematically assess whether underlying statistical assumptions are fulfilled for all statistical analyses.

This statistical analysis plan also has limitations. Our primary conclusions will be based on the main outcome, while we will also evaluate secondary outcomes, exploratory outcomes, and subgroup analyses. We have not adjusted our significance thresholds for all comparisons, acknowledging that this approach increases the risk of type I errors. Additionally, the anticipated intervention effects, which informed our sample size and power calculations for the secondary outcomes, are based on a pragmatically applied identical intervention effect across all three trials using clinical judgment and results from earlier similar trials. This anticipated effect is deemed both realistic and of significant value to patients. When drawing final conclusions, we will consider the elevated risk of type I errors and uncertainties surrounding the anticipated intervention effects.

## 4 CONCLUSION

Here we describe the statistical analyses for the STEPCARE trial in detail. The aim of this predefined statistical analysis plan is to minimize the risk of analysis bias.

## AUTHOR CONTRIBUTIONS

CBK, JD, MBS, NN, and JCJ wrote the original draft. All authors critically reviewed and approved the final manuscript.

## FUNDING INFORMATION

This work has been funded by The Swedish Research Council, ALF‐project funding within the Swedish Health Care, The Academy of Finland, Sigrid Jusélius Foundation (Finland), Hospital District of Helsinki and Uusimaa (Finland), Medicinska Understödsföreningen Liv och Hälsa (Sweden), Medical Research Future Fund (Australia), Health Research Council of New Zealand, and Clinical Research Programme Directorate of Health Ministry of Health and Social Security (Luxembourg).

## CONFLICT OF INTEREST STATEMENT

Pellis T. received lecture fees from BD. Leithner C received research support from Laerdal Foundation. Glover G received consulting and speaker fees from Sedana Medical; BD; and research grants from Mindray and Sedana Medical. Nee J received honorarium and travel costs for presentations from BD BARD and Xenios AG. Pareek N received Abbott Vascular—Honoraria, Consultancy, Educational Grants, and NIPRO—Honoraria, Shockwave Medical—Consultancy. Skrifvars M received a speaker's fee from BARD Medical (Ireland) 2022 and he is a member of the editorial board of Acta Anaesthesiologica Scandinavica.

## Data Availability

Not applicable. All available data are included in this manuscript.
